# Effect of Plasma-Treatment of Interleaved Thermoplastic Films on Delamination in Interlayer Fibre Hybrid Composite Laminates

**DOI:** 10.3390/polym12122834

**Published:** 2020-11-28

**Authors:** Salvatore Giacomo Marino, Florian Mayer, Alexander Bismarck, Gergely Czél

**Affiliations:** 1 Department of Polymer Engineering, Faculty of Mechanical Engineering, Budapest University of Technology and Economics, Műegyetem rkp. 3, H-1111 Budapest, Hungary; marinos@pt.bme.hu; 2 Polymer and Composite Engineering (PaCE) Group, Institute for Materials Chemistry & Research, Faculty of Chemistry, University of Vienna, Währingerstrasse 42, A-1090 Vienna, Austria; f.mayer@univie.ac.at (F.M.); alexander.bismarck@univie.ac.at (A.B.); 3 Department of Chemical Engineering, Imperial College London, South Kensington Campus, London SW7 2AZ, UK

**Keywords:** fibre hybrid composites, fracture toughness, delamination, film-interleaving, plasma-treatment

## Abstract

Safe, light, and high-performance engineering structures may be generated by adopting composite materials with stable damage process (i.e., without catastrophic delamination). Interlayer hybrid composites may fail stably by suppressing catastrophic interlayer delamination. This paper provides a detailed analysis of delamination occurring in poly(acrylonitrile-butadiene-styrene) (ABS) or polystyrene (PS) film interleaved carbon-glass/epoxy hybrid composites. The ABS films toughened the interfaces of the hybrid laminates, generating materials with higher mode II interlaminar fracture toughness (*G_IIC_*), delamination stress (*σ_del_*), and eliminating the stress drops observed in the reference baseline material, i.e., without interleaf films, during tensile tests. Furthermore, stable behaviour was achieved by treating the ABS films in oxygen plasma. The mechanical performance (*G_IIC_* and *σ_del_*) of hybrid composites containing PS films, were initially reduced but increased after oxygen plasma treatment. The plasma treatment introduced O-C=O and O-C-O-O functional groups on the PS surfaces, enabling better epoxy/PS interactions. Microscopy analysis provided evidence of the toughening mechanisms, i.e., crack deflection, leading plasma-treated PS to stabilise delamination.

## 1. Introduction

Delamination can lead to severe damage in composites and compromise the load-bearing capacity of the material [1,2]. It is one of the reasons why metals are not replaced with composites in several industrial applications. The designers’ efforts to make fibre reinforced polymer composites (FRPC) more delamination-resistant mainly focus on improving interlaminar fracture toughness, which is the material’s ability to resist interlaminar crack propagation, i.e., delamination. In practical situations, delamination may propagate under different modes of deformation, for instance by opening (mode I), shearing (mode II) and in mixed mode. Our study focuses on delamination in pure mode II. Studies on mode II interlaminar fracture toughness (*G_IIC_*) in composites have highlighted the difficulty of developing a simple and widely accepted test to determine the mode II energy release rate *G_II_* in the material under load [3].

Delamination and the evaluation of *G_IIC_* deserve the attention of researchers as a thorough understanding of this phenomenon may help engineers to design safer composite components for practical applications. Hybrid composites have the potential to be safer than conventional FRPCs [4,5,6], and show pseudo-ductility as reported in recent studies [5,7,8]. Fibre hybrid composites have two types of fibre reinforcements with different strains to failure, e.g., carbon and glass fibres, embedded in a common matrix system. Studies on fibre hybrid composites usually distinguish the reinforcements by their strain to failure: e.g., low-strain and high-strain materials (LSM and HSM), respectively. Pseudo-ductile hybrid composites are attractive because hybridisation adds a stable, rising plateau (non-linear stage) to the usually linear tensile stress–strain response of composites. The detectable stress plateau of pseudo-ductile hybrid composites generates a wide safety margin before the final failure of the material, in contrast to traditional composites, which fail abruptly [9]. Czél et al. in [7] demonstrated that sufficient *G_IIC_* is one of the key parameters in achieving stable damage modes in interlayer fibre hybrid composites by the suppression of catastrophic delamination at the first fracture of the thin LSM layer and enabling its fragmentation process. Therefore, hybrid composites benefit from increased *G_IIC_*, which may help to suppress delamination and stabilise fragmentation of thicker LSM layers.

The most used techniques to make FRPCs tougher reported in literature were reviewed in [10,11,12,13,14]. Furthermore, studies [11,15,16,17,18,19] on toughening FRPCs have shown benefits from mixing thermoset (TS) matrices, e.g., epoxy resins, with thermoplastic (TP) or rubber particles. Rubber-toughened matrices undergo phase separation between the two constituents, creating finely dispersed rubber-rich domains well bonded to the epoxy matrix. Generally speaking, a higher interlaminar fracture toughness is often associated with the introduction of new energy absorption mechanisms, i.e., crack deflection and arrest, which make the composite more delamination resistant, as also reported in [20,21,22,23]. Thermoplastic matrices offer greater toughness than thermoset materials [24,25], but they generally have inferior mechanical properties, thermal stability and adhesion to fibres than thermoset matrices [26]. Thermoplastic film interleaving is a simple practical approach to modifying fibre-reinforced thermoset composite interfaces for superior toughness compared to the reference materials without interleaves [27,28]. Interleaving composites with thin, flexible thermoplastic films increases their tolerance to delamination through crack arresting and deflection mechanisms if the TP films bond well to the composite layers [27,29,30,31,32,33,34]. Processing the interleaved composites at temperatures that soften the TP interleaf films can enhance their interaction with the TS matrix system of the composite plies. The film interleaving technique is particularly suitable for high-performance composites, which are usually fabricated from prepregs [27,28] and the extra thermoplastic film layers are simple to integrate into industrial manufacturing processes.

The benefits of film interleaving may be enhanced by engineering surface activation, e.g., plasma treatment, which can improve the wettability of thermoplastic films and promote better adhesion to a thermoset matrix of composite layers [35,36,37,38,39,40,41]. The plasma treatment affects the morphology and chemical character of polymeric film surfaces through the exposure to free radicals, electrons and ions in the plasma. The UV radiation during low-pressure plasma treatments particularly facilitates the scission of the polymer chains, the crosslinking or the activation of the polymer surface by introducing functional groups through the interaction between radicals in the plasma and on the polymer surface [35,42]. 

J. Parameswaranpillai et al. [43] summarised the literature which indicates that thermoplastic polymers, such as polysulfone (PSF), poly(ether sulfone) (PES), and poly(ether imide) (PEI), are suitable additives for introducing toughening effects in epoxy/TP systems. The disadvantage of using these thermoplastics is that high temperatures or additional post-curing cycles are required for curing the modified laminates, due to the high softening temperature of the interleaves (their glass transition temperature (*T_g_*) is higher than 200 °C).

The aim of this study was to achieve stable pseudo-ductile failure in interlayer fibre hybrid composites made with standard thickness LSM by improving the *G_IIC_* of the interfaces within the laminates. To this end, we studied mode II delamination in carbon/S-glass fibre epoxy hybrid composites and explored the toughening effect of interleaving thin thermoplastic films of poly(acrylonitrile-butadiene-styrene) (ABS) and polystyrene (PS) on delaying, or suppressing, delamination. The selected thermoplastics had lower *T_g_* than the curing temperature recommended for the epoxy system used here (i.e., 125 °C), thus processing conditions enabling good integrity of the layers were provided. We also exposed the selected thermoplastic films to low-pressure oxygen plasma treatment to increase their hydrophilicity through the formation of functional groups oxygen containing, promoting strong bonding to the epoxy matrix system of the composite layers. It is also worth noting that the applied composite prepreg plies were not special thin types as those used in earlier demonstrative studies [7,9] but standard thickness, commercially available materials available at moderate price, making film interleaved hybrid composites a more accessible and feasible option for industrial applications.

## 2. Materials and Methods 

### 2.1. Materials for Fibre-Hybrid Composites

**Figure 1:**
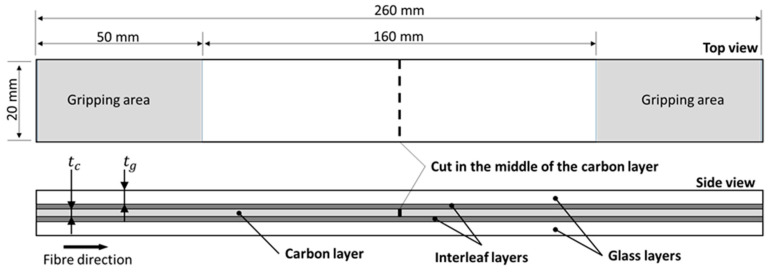
Geometry and dimensions of the specimens. The grey-coloured areas in the top view are the gripping areas covered by sandpaper. The side view shows the layup structure of the interleaved hybrid composite. The baseline configuration follows the same structure, without the interleaf layer.

The laminates were made by hybridising prepregs of HexTow^®^ IM7 carbon and AGY S-2 glass fibres as shown in Figure 1 schematically. The prepregs were manufactured with the same epoxy system, i.e., HexPly 913 supplied by Hexcel in 300 mm wide rolls. The mechanical properties of the dry fibres and the nominal properties of the cured prepreg plies are reported in Table 1 and Table 2.

### 2.2. Intrerleaved Thermoplastic Films

The thermoplastic films interleaved in hybrid composites were Starex^®^ LX0981 poly(acrylonitrile-butadiene-styrene) (ABS) (Lotte Chemical, South Korea) and Empera^®^ polystyrene (PS) (Styrolution, Germany). The ABS films were produced by pellet extrusion (previously dried for 4 h at 80 °C as indicated in the manufacturer’s datasheet) on a LABTECH Scientific 25–30 + LCR300 extruder equipped with a 25 mm diameter single screw and a flat film die. The length/diameter ratio of the screw was l/d = 30. The barrel temperatures were set in the range of 210 to 225 °C, and the die was tempered to 230 °C. The thickness and the usable width of the films was 40 µm and 180 mm, respectively. The PS film was kindly provided by TCKT (Wels, Austria) as a 40 µm film in 160 mm width. The mechanical and thermal properties of the thermoplastics used are reported in Table 3.

### 2.3. Oxygen Plasma Treatment of Interleaf Films

The PS and ABS films were treated in oxygen (O_2_) plasma to activate their surfaces for better bonding to epoxy. We treated the films at 25 °C for 2 and 4 min, to investigate the effect of exposure time as well. The gas flow rate in the plasma reactor (Diener Electronic, Germany) was set to 50 sccm (standard cm^3^/min) and the power was 50 W. The chamber of the reactor was purged with oxygen before igniting the plasma. It was evacuated to 0.1 mbar and filled with oxygen up 0.35 mbar, then purged. The filling/purging was repeated three times to reduce external contaminators in the chamber.

### 2.4. Surface Analysis of Plasma-Treated Polymer Films by X-ray Photoelectron Spectroscopy

The elemental composition of both ABS and PS samples before and after plasma treatment was analysed via X-ray photoelectron spectroscopy (XPS). For each sample, a survey spectrum with a step size of 1 eV as well as element specific spectra for Carbon (279–298 eV) and Oxygen (525–545 eV) with step sizes of 0.1 eV were recorded. The measurements were performed with Al-Kα X-rays and a spot size of 400 µm on a Thermo Scientific Photoelectron Spectrometer (Nexsa, UK). We performed a second set of measurements for each sample after 120 s surface ablation with low energy Ar clusters (6000 eV, 1000 atom clusters) to determine if the plasma treatment penetrated below the top layers of the polymer films.

### 2.5. Contact Angle Measurements

The water contact angle of untreated and plasma-treated thermoplastic films was determined with a DSA-30S type Drop Shape Analyser (Krüss, Germany). The whole process was automatic and controlled by the software *Advanced*. We used a delay of 5 min between the plasma treatment of the thermoplastic films and the first measurement, to take into account of the time necessary to put the treated films in the prepregs during composite manufacturing. The water contact angle was measured on untreated and plasma-treated ABS and PS samples for at least 100 droplets, and the resulting values were averaged.

### 2.6. Design and Manufacturing of Hybrid Composite Laminates

Hybrid composites were manufactured from carbon/epoxy and glass/epoxy prepregs. The stacking sequence used was [G_2_/F/C/F/G_2_] (see the schematic in Figure 1), where G stands for glass/epoxy plies, C for one carbon/epoxy ply and F for interleaf film layers. The subscript numbers indicate the number of plies used in each glass/epoxy layer. The laminate configuration used in this paper was designed to (1) promote delamination between the carbon/epoxy and the glass/epoxy layers, without encountering fractures of the carbon layer, and (2) avoid premature failure of the laminates before complete delamination during the tensile tests (i.e., to prevent the fracture of the glass/epoxy layers before complete delamination). For the first criterion, we designed hybrid composites in a way to get pure delamination and avoid fractures of the carbon layer. Delamination, in continuous hybrid composites, occurs if mode II fracture toughness (*G_IIC_*) of the laminate is lower than the mode II energy release rate (*G_II_*) at the first fracture of the carbon/epoxy layer [44]. The estimated *G_IIC_* of our carbon/glass-epoxy hybrid composites is in the range of 1.0 to 1.5 kJ/m^2^ based on previous measurements on similar materials and laminate configurations. The *G_II_* of a given material combination with a specific laminate configuration can be calculated with Equation (1) [45].

(1) $$G_{IIC}<G_{II}=\;\frac{\varepsilon_{f,c}^{2}E_{c}t_{c}\left(2E_{g}t_{g}+E_{c}t_{c}\right)}{8E_{g}t_{g}}$$

where $\varepsilon_{f,c}$ is the tensile strain to failure of the carbon fibres, $E_{g}$ and $E_{c}$ are the tensile moduli of the glass and the carbon layers, respectively; $t_{g}$ and $t_{c}$ are the thicknesses of the glass/epoxy and the carbon/epoxy layers respectively. The configuration with double glass/epoxy plies next to the central carbon ply yields a *G_II_* of 3.3 kJ/m^2^, which is well above the estimated *G_IIC_* range. Therefore, delamination of the layers is expected_._

To satisfy the second criterion, we calculated the minimum thickness of the glass/epoxy layers with inequality (2) [45] to withstand the full load on the specimen at the first fracture of the carbon layer.

(2) $$t_{g}>\;\frac{\varepsilon_{f,c}E_{c}t_{c}}{2E_{g}\left(\varepsilon_{f,g}-\varepsilon_{f,c}\right)}$$

The minimum required thickness (*t_g_*) for one glass/epoxy layer is 0.279 mm (the thickness of two glass/epoxy plies is 0.308 mm), therefore we do not expect premature failures in the configuration here adopted for the baseline laminate with two glass/epoxy plies for each glass layer, i.e., [G_2_/C/G_2_]. We adopted the laminate architecture introduced by Wisnom [46] and Cui et al. [47] in their studies on delamination in FRPCs and measured the *G_IIC_* in the investigated hybrid configurations by simple tensile tests. This architecture consisted of a unidirectional (UD) pre-cut LSM layer inserted between two continuous HSM layers of UD fibre-reinforced plies, to form a symmetrical layup with a discontinuity in the middle layer transversal to the fibre orientation to induce shear stresses between the layers. This architecture helps delamination to start early so that it can be studied. The cut was made in the middle of the carbon/epoxy layer, perpendicular to the fibre direction, as shown in Figure 1. A rotary cutter with a 25 mm diameter blade was used to make the cuts and reduce the possible shearing effect introduced to the fibres by cutting prepregs with a simple V shape blade. The laminates were placed in a vacuum bag and cured in a hot press (Carver, Inc., USA) at 125 °C for 60 min. The pressure applied on the laminates was 0.7 MPa. The cured plates were cut with a diamond wheel cutter. The final size of the samples was 260 mm × 20 mm.

### 2.7. Mechanical Testing Methods and Calculation of the Parameters

The specimens were tensile tested under uniaxial tensile loading with controlled displacement and at a crosshead speed of 2 mm/min with a universal electro-mechanical testing machine (5969 type 50 kN rated Instron, UK) equipped with wedge type mechanical grips. Strains were measured with an iMETRUM video extensometer system. The number of samples tested for each material configuration was dependent on the quality of the cured plates and it varied from three to five samples. As the glass/epoxy layers were placed to the outside parts of the hybrid laminates, it was possible to test the specimens without end-tabs as the focus of the study was the analysis of delamination and not the final failure strain of the laminates, which were still affected by stress concentration in the S-glass/epoxy layers at the grips. The ends of the specimens were covered with 50 mm long P80 grit size sandpaper pieces with the rough side turned to the specimen surface to protect them from damage from the sharp nails of the grip faces, but sufficient friction was maintained so as not to let the specimens slide out during loading (see Figure 1). 

**Figure 2:**
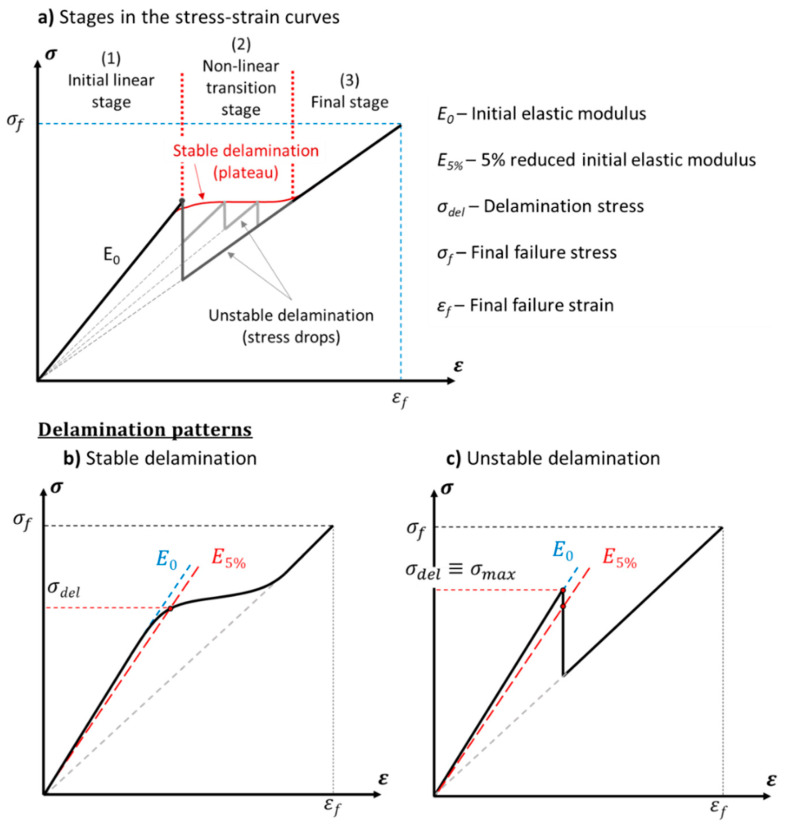
(**a**) Qualitative tensile stress-strain curves of the laminates and schematics of the (**b**) stable and (**c**) unstable delamination patterns.

The tensile stress in the samples was calculated with their nominal thickness to make the different configurations comparable to each other and avoid discrepancies in the results caused by potential thickness variations caused by uneven resin leakage during hot pressing. Furthermore, the thickness of the interleaves was not considered in the calculation as the normal stress carried by them is negligible in comparison with that taken by the fibre reinforced layers (i.e., two orders of magnitude lower). The initial tensile modulus and the delamination stress are schematically defined in Figure 2. In particular, the delamination stress was defined according to the possible delamination patterns occurring during the tests. The identified scenarios are schematically depicted in Figure 2b,c and labelled as *stable* and *unstable* cases. The *stable* case shows a delamination mode characterised by a smooth transition between the initial linear stage and the plateau developed by the stable delamination of the composite layers. This delamination pattern is characterised by slow growth-rate delamination starting from the discontinuity in the carbon/epoxy layer. The tensile stress–strain curve of the laminate progressively deflects from the initial linear trend as soon as delamination becomes extensive in the material [3]. Delamination starts to be highly sensitive to a further increase of stress at the onset of delamination, which is identified here with the intersection of the tensile stress–strain curve with the line representing a 5% laminate stiffness reduction, due to delamination ($E_{5\%}$). This way, we defined the delamination stress ($\sigma_{del}$) in the case of a *stable delamination* pattern. The *unstable* case is characterised by a sudden stress drop interrupting the initial linear stage. Here, the intersection between the stress–strain curve and the 5% reduced initial tensile modulus line is below the maximum stress reached before delamination has spread. In this case, $\sigma_{del}$ is defined as the local maximum stress ($\sigma_{max}$) as it represents the maximum load the sample can withstand before the sudden propagation of delamination. The mode II interlaminar fracture toughness is calculated with Equation (3) [7]:

(3) $$G_{IIC}=\;\frac{\sigma_{del}^{2}h^{2}E_{c}t_{c}}{8E_{g}t_{g}\left(2E_{g}t_{g}+E_{c}t_{c}\right)}$$

where $\sigma_{del}$ is the delamination stress (see Figure 2) and h the full thickness of the laminate (excluding the thickness of the interleaved films; then *h = 2t_g_ + t_c_*). The glass/epoxy and carbon/epoxy layer thicknesses of the layers, i.e., *t_g_* and *t_c_*, respectively, are based on the number of plies and the nominal ply thicknesses given in Table 2.

### 2.8. Microscope Analysis

Scanning electron microscope (SEM) analysis of the delaminated samples was performed with JEOL JSM 6380 LA equipment (Jeol Ltd., Japan). The samples were sputter-coated with gold to avoid static charging during imaging. The digital microscope analysis was conducted with a Keyence VHX-5000 digital microscope (Keyence Corp., Japan). 

## 3. Results and Discussion

### 3.1. Effect of Plasma Treatment on the Wettability of the Applied Polymer Films

The plasma treatment made the previously hydrophobic surfaces of both thermoplastics more hydrophilic. After 2 min low pressure O_2_-plasma treatment, the water contact angles dropped from 93° to 35° for ABS, and from 85° to 22° for PS films, respectively (Table 4) showing the significantly improved wettability of the treated films. For ABS films, extending the plasma treatment time to 4 min did not significantly improve wettability. However, after the PS films were exposed to the low-pressure O_2_-plasma for 4 min, the water droplets spread on the films, indicating complete wetting, i.e., a contact angle of 0°. For further testing, we only used the thermoplastic films plasma-treated for 2 min as interleaves to produce hybrid composites.

### 3.2. XPS Analysis

We analysed the surfaces of PS and ABS films by X-ray photoelectron spectroscopy to gather information of their elemental composition and the bond character of the oxygen in their surfaces. The analysed configurations of both film materials were the following: (i) untreated, (ii) untreated + argon ablated, (iii) plasma-treated and (iv) plasma treated + argon ablated. The surface analysis allowed us to check if the plasma treatment of the polymer films introduced the functional groups capable to promote covalent bonds to the epoxy contained in the fibre-reinforced layers.

#### 3.2.1. Determined Elemental Surface Composition 

The untreated ABS film had a surface oxygen content of 5.3 at % (i.e., atomic %), which came from the absorption of adventitious carbon species (i.e., carbon that is adsorbed to the surface from the environment during production, handling or storage of the films). The oxygen content was increased to 17.3 at % by the plasma treatment showing that the treatment successfully introduced oxygen-containing functional groups to the surface. The elemental composition analysis after argon ablation (i.e., removing the top atomic layers) of both untreated and plasma-treated ABS films, revealed that the oxygen content dropped to negligible values of around 1 at %, as shown in Table 5, indicating pristine ABS for both the modified and unmodified sample [48]. This shows that the plasma treatment only affected the surface layer of the ABS.

The untreated PS film had a surface oxygen content of 5.8 at %, which also stemmed from environmental contamination. The elemental composition of the PS film surfaces changed significantly after plasma treatment (see Table 5). The oxygen plasma-treated PS films had an oxygen content of 21.3 at %, significantly higher than that of the untreated sample, confirming successful surface modification. In the bulk volume (i.e., after argon ablation) the elemental composition of the plasma-treated and untreated PS was identical and consisted mainly of carbon (>99.5%) and negligible amounts of oxygen for both samples, as it is expected for pristine PS. 

The data related to the bulk phase, i.e., after ablation, of both TP materials (ABS and PS) shows that any environmental contamination (in case of the untreated samples) as well as the surface modification introduced during plasma treatment was removed, and therefore the data can be considered truly representative of the elemental composition of the pristine materials. In the next paragraph, we will compare the effect of the plasma treatment to the pristine (argon ablated) ABS and PS.

#### 3.2.2. Analysis of the Bonding Mechanism of Oxygen on the Treated Polymer Surfaces

**Figure 3:**
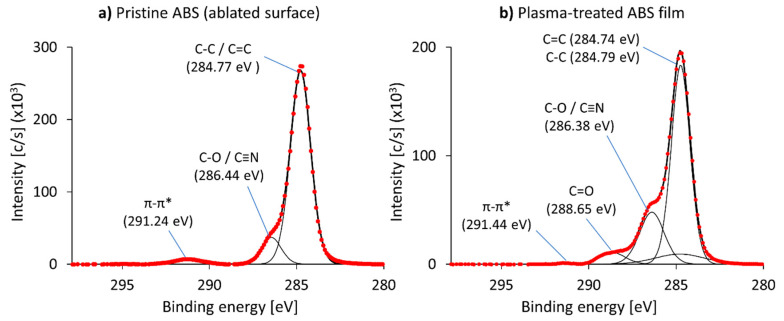
Background corrected C1s spectra of (**a**) pristine (after Ar cluster surface ablation) and (**b**) plasma-treated ABS. The measured spectrum is depicted as points, whereas the envelope and the deconvolution thereof are depicted as solid lines. (Please note that the scale of the vertical axes is different in the two graphs as we wanted to compare the C=C peaks. This way, the areas of the two C1s spectra are visually comparable, too).

**Figure 4:**
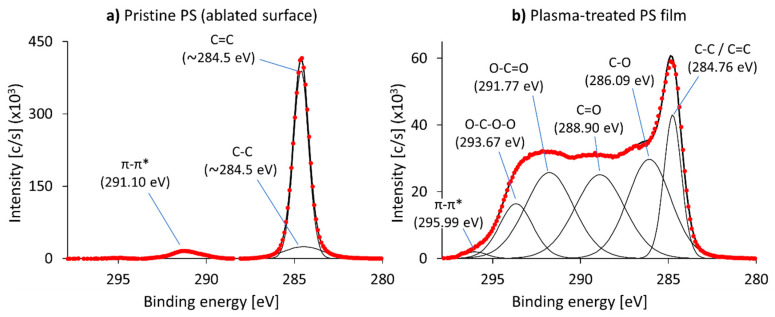
Background corrected C1s spectra of (**a**) pristine (after Ar cluster surface ablation) and (**b**) plasma-treated PS. The measured spectrum is depicted as points, whereas the envelope and the deconvolution thereof are depicted as solid black lines. (Please note that the scale of the vertical axes is different in the two graphs as we wanted to compare the C=C peaks. This way the areas of the two C1s spectra are visually comparable, too.).

With the C1s spectra plotted in Figure 3 and Figure 4, we compared the bonds detected in the pristine ABS and PS films with the bonds generated by oxidation during the plasma treatment. The main difficulty with this process was that the peaks of the plasma-treated films were broad and shifted to higher energy levels due to sample overcharging. Therefore, we will limit our discussion to a qualitative analysis of the XPS results. The carbon specific elemental spectrum (C1s spectrum, i.e., the binding energy of the electrons of the carbon atom’s 1 s orbital) reported in Figure 3a is related to the pristine ABS film (ablated surface).

A large carbon-carbon peak at 284.8 eV was observed, which was a result of the overlapping peaks of C-C and C=C bonds and a feature at +1.67 eV (286.4 eV) caused by an overlap of the C≡N of the polymer and possibly a small amount of residual C-O groups. The π-π* stacking feature (caused by the delocalization of the electrons in the aromatic rings of the system) was also present at +6.47 eV (291.2 eV). The C1s spectrum related to the plasma-treated ABS film (see Figure 3a) was well fitted by a C=C component at 284.7 eV, C-C at +0.05 eV (284.8 eV), C=O at +3.91 eV (288.7 eV) and a satellite peak of π-π* stacking feature at +6.7 eV (291.4 eV) as well as a combined feature of the overlapping peaks of C-O and C≡N bonds at +1.64 eV (286.4 eV). We found that the placement of these peaks was in accordance with previously published research for ABS [48]. The results show that the plasma treatment only introduced hydroxyl groups (the C-O/C≡N peaks after plasma modification were larger than without plasma treatment compared to the respective C-C/C=C peak) and carbonyl groups (a C=O peak is present after modification but not in the ablated sample). While the presence of these functional groups increases the surface polarity and energy, and therefore the hydrophilicity and wettability of the samples, as shown above by the results of the contact angle measurements, they cannot react chemically with the epoxy matrix, and no covalent bonds between the ABS sheets and the epoxy resin should form. Then, we do not expect a significant improvement of the interfacial mechanical properties in the hybrid composites containing plasma-treated ABS films. 

The deconvolution of the C1s spectrum related to the pristine PS film is shown in Figure 4a. We registered carbon-carbon bonds (C-C and C=C) at ~284.5 eV, caused by the aliphatic and aromatic components of the polymer, and a π-π* stacking feature at +6.6 eV (291.1 eV). The deconvolution of the C1s spectrum of the plasma-treated PS film (see Figure 4b) was fitted reasonably through a C-C/C=C component at 284.8 eV, C-O at +1.33 eV (286.1 eV), C=O at +4.1 eV (288.9 eV), O-C=O at +7.01 eV (291.8 eV), O-C-O-O at +8.9 eV (293.7 eV) and a π-π* stacking feature at +11.23 eV (296.0 eV). These features were in accordance with a previous study [49] albeit shifted further. We suspected that this was caused by the harsher plasma treatment, as both the treatment time was longer, and the oxygen content of the plasma was higher than in the previously mentioned publication, leading to a more heavily modified surface. 

Similarly to ABS, the plasma treatment of PS introduced hydroxyl and carbonyl groups, as well as peroxide (O-C-O-O) and carboxylic (O-C=O) groups. This drastically increased hydrophilicity as well as wettability of the PS surfaces. This was previously confirmed by the measured contact angles and lead us to expect a better compatibility between the surface of the PS films and epoxy. Furthermore, the introduction of carboxylic and peroxide groups allows for chemical bonding between those groups and the epoxide function of the epoxy resin because both of these groups can act as nucleophiles similar to the amine functional groups of the hardener component of epoxy systems. A covalent bond could possibly form during the processing of the prepreg plies and the plasma-treated PS films, which may lead to a significantly stronger adhesion between them.

### 3.3. Discussion of the Mechanical Test Results

#### 3.3.1. Delamination in the Baseline Hybrid Composites

**Figure 5:**
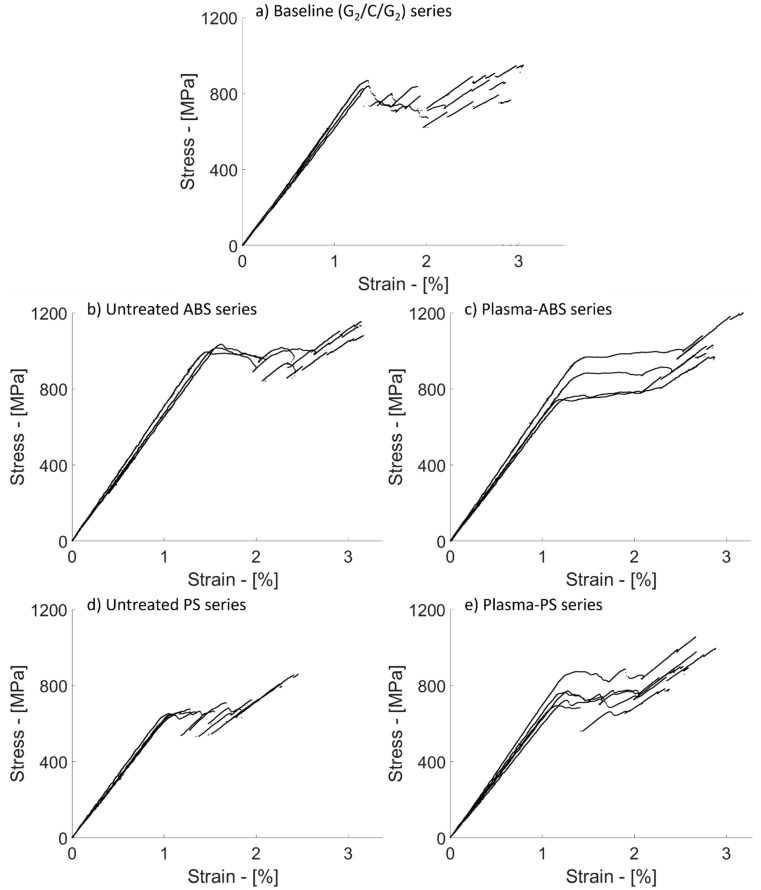
Stress-strain curves of tensile tests on the following hybrid composite configurations: (**a**) Baseline [G_2_/C/G_2_]; (**b**) ABS and (**c**) plasma-treated ABS film-interleaved laminates; (**d**) PS and (**e**) plasma-treated PS film-interleaved laminates.

**Figure 6:**
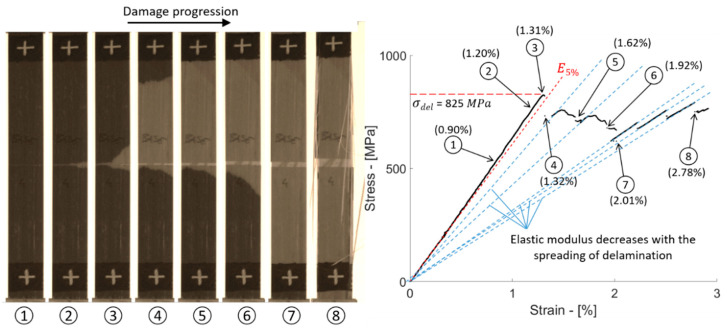
Stages of damage progression in a baseline sample [G_2_/C/G_2_]. The image shows the progressive spread of delamination with the increasing strain acting on the sample.

The delamination resistance of the baseline configuration, i.e., [G_2_/C/G_2_], was evaluated through tensile tests. The resulting stress–strain curves are plotted in Figure 5a. At the initial linear stage of the tests, the samples exhibited an elastic modulus of 64.2 GPa. Table 6 reports the mechanical parameters of the *baseline series* calculated with the nominal and measured thickness of the samples. The initial linear stage was interrupted by stress drops due to the sudden spread of delamination. The delamination produced light zones well visible from the outer surfaces of the specimens in contrast to the darker undamaged parts (see damage sequence in Figure 6). This feature is due to the translucency of the glass/epoxy plies and it was already observed in other studies on carbon/glass–epoxy hybrid composites [50,51]. 

The sample investigated in Figure 6 shows the steps of damage evolution in detail. Delamination started from the pre-cut line in the carbon/epoxy layer, well visible at around 0.9% strain (stage 2). At a well visible delaminated area, we observed a deflection in the stress–strain curve from the initial linear trend (see stage 3), just before the significant stress drop due to the catastrophic spreading of delamination affecting about one third of the full specimen area (stage 4). Delamination continued to propagate causing a number of limited stress drops in the stress–strain graph (stages 5–7). 

We observed that delamination first spread in one half of the sample (i.e., above or below the pre-cut line in the carbon/epoxy layer) and continued to the opposite side only after complete separation of the first side (see stages 4–6). The delamination finished at around 2% strain (see stage 7), and the sample continued to take further stress without the contribution of the carbon/epoxy layer, in the final stage, where splitting in the glass/epoxy layer occurred (see stage 8). The blue dashed lines in the graph (Figure 6) show the correlation between the spread of delamination in the sample and the reduction of its elastic modulus. After the drops in the stress–strain curve due to delamination, the sample was re-loaded following a new linear trend whose slope was lower than $E_{0}$, the initial elastic modulus. With the sample shown in Figure 6, we also wanted to show that the initial stage of delamination around the pre-cut line in the carbon/epoxy layer (i.e., between stages 2 and 3) did not affect the initial linear trend in the stress–strain curve ($E_{0}$) significantly. Delamination became highly sensitive to a further increase of stress and propagated suddenly at the registered value of 825 MPa, i.e., delamination stress, used here for the calculation of the $G_{IIC}$ of the sample. The average delamination stress for the baseline series, calculated with the method introduced in Figure 2, was around 845 MPa and the resulting $G_{IIC}$ was equal to 1.503 kJ/m^2^ on average (Table 6). 

**Figure 7:**
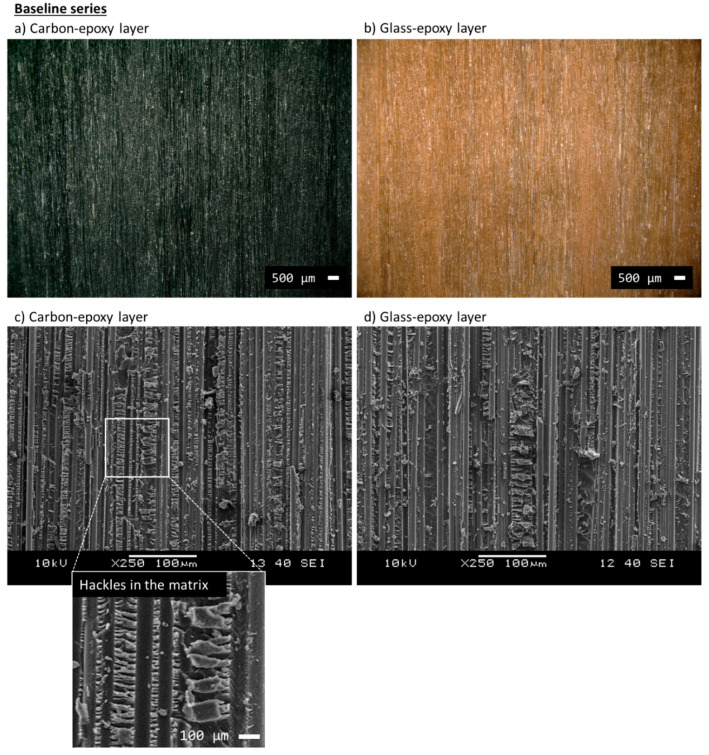
Microscope analysis of the baseline samples [G_2_/C/G_2_] after delamination: (**a**) carbon/epoxy layers analysed with digital microscope and (**c**) SEM; (**b**) glass/epoxy layers analysed with digital microscope and (**d**) SEM. The inset in (**c**) provides a detailed view of the hackles of the epoxy matrix.

The microscope analysis performed with the digital and scanning electron microscopes can be seen in Figure 7. Images (a) and (b) were taken with the digital microscope and are useful to record the overall appearance (colour, continuity, texture etc.) of the carbon/epoxy and glass/epoxy surfaces after delamination. Through the SEM analysis in (c) and (d), we checked the fracture mechanisms at a smaller scale. The carbon/epoxy and glass/epoxy layers showed fibres surrounded by matrix, which failed leaving a massive presence of epoxy hackles (see the inset in (c) for a detailed view). These hackles are typical of shear (mode II) deformation and failure [52,53].

#### 3.3.2. Hybrid Composites Containing ABS Film Interleaves 

**Figure 8:**
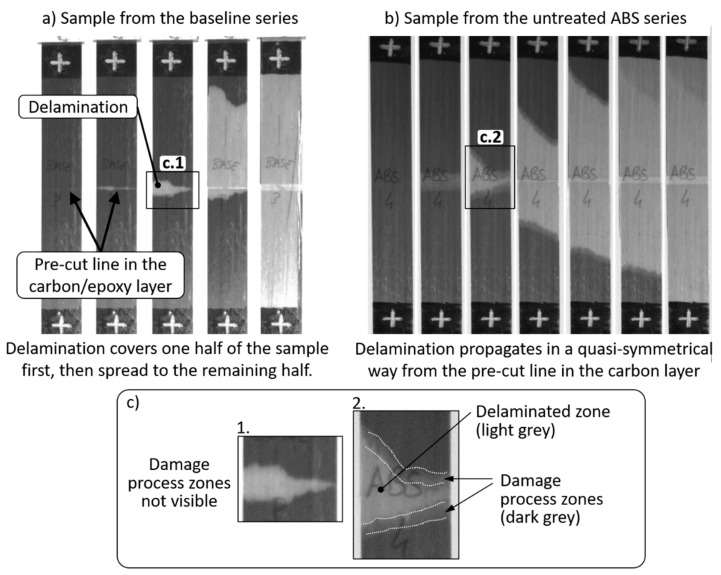
Comparison between the delamination patterns of samples from the (**a**) baseline and the (**b**) *untreated ABS series*. Part (**c**) shows magnified images of the pre-cut regions where delamination initiates from.

The tensile tests on hybrid composites containing ABS film interleaves generated the stress–strain curves plotted in Figure 5b. The resulting mechanical properties of the *untreated ABS series* were better than the ones measured in the baseline samples, i.e., +20% for delamination stress and +44% for the $G_{IIC}$. Even if $\sigma_{del}$ is calculated with the measured thickness, it is 5% higher than in the case of the baseline series (see Table 6). The presence of the ABS films affected the propagation of delamination in the samples, delaying and making it more stable in comparison with the baseline. The stress–strain curves of the *untreated ABS series* were characterised by stress drops with low intensity, in contrast to the *baseline series* (see Figure 5b). Delamination started from the pre-cut line in the carbon/epoxy layer and symmetrically spread in both directions of the samples with a slow growth rate. In contrast, the *baseline series* was characterised by a sudden delamination in one half of the samples first and, subsequently, propagation in the remaining half (see the comparison in Figure 8). 

Also, from observations made on the video frames captured during the tensile tests (see Figure 8b), the *untreated ABS series* showed two zones in the proximity of the crack front, distinguishable by their grey level, as shown in Figure 8c in detail. It is reasonable to consider that the medium dark area around the delamination crack fronts are the process zones of the cracks, whereas in the light grey area, the layers are fully separated. As shown in Figure 8c, this phenomenon was not observed in the baseline samples, which did not contain TP interleaf layers taking part in the delamination process. Based on our visual observations it is reasonable to assume that we detected the damage process zone, i.e., an extended plasticised part of the ABS film in front of the delamination crack front. Possible reasons for slow and stable mode II delamination crack propagation in the *untreated ABS series* are the longer damage process zone which reduced stress-concentration around the crack tips and the presence of the rather soft ABS layer, which could have deformed plastically and blunted micro-cracks. We also hypothesise that the presence of the ABS films well bonded to the composite plies and possibly capable of plastic deformation were able to distribute the high stresses at the delamination crack tips to larger zones even though their strength was inferior to epoxy. This way, a weaker, but more plastic interlayer was successful in stabilizing and suppressing the delamination process. 

**Figure 9:**
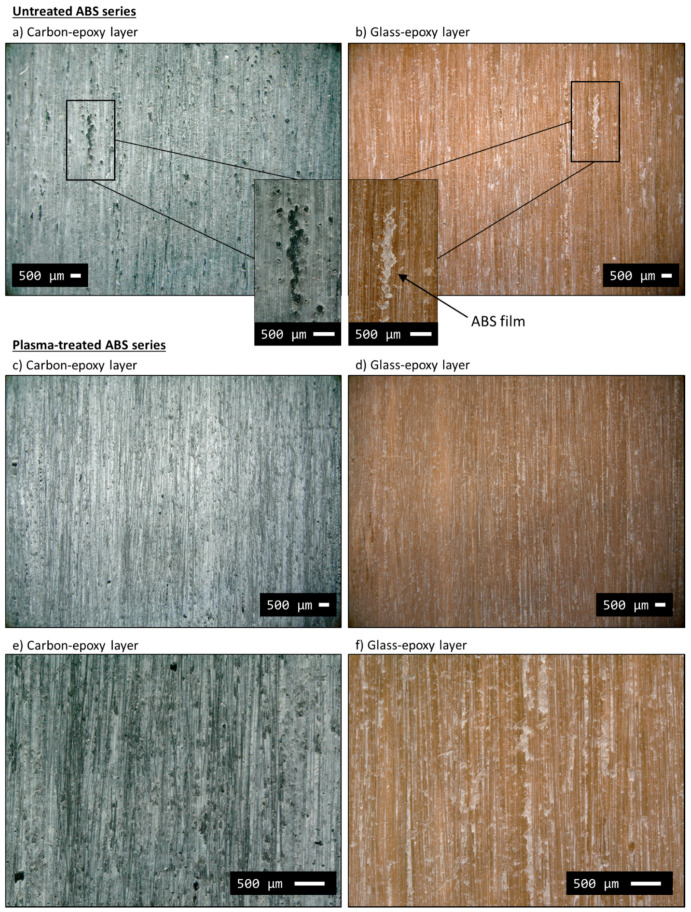
Delaminated surfaces of typical samples from the ABS (**a**,**b**) and the plasma-ABS series (from (**c**–**f**)) analysed with digital microscope.

**Figure 10:**
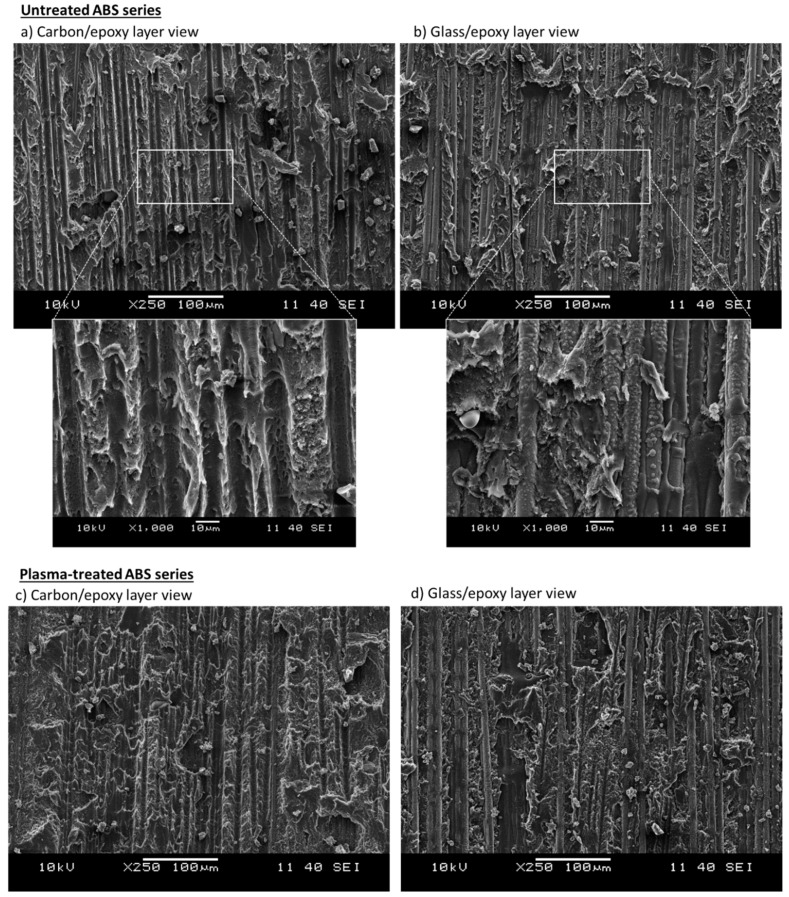
Delaminated surfaces of typical specimens from the ABS (**a**,**b**) and the plasma-ABS ((**c**,**d**)) series analysed with SEM.

**Figure 11:**
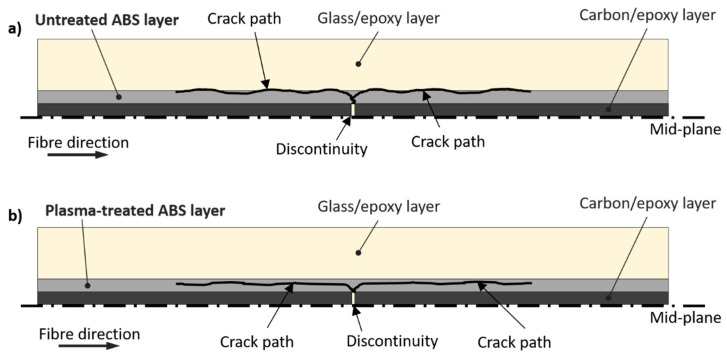
Representation of the crack path in ABS-interleaved hybrid composites (**a**) with untreated ABS (**b**) with plasma-treated ABS.

The images shown in Figure 9a,b from digital microscope analysis show both carbon/epoxy and glass/epoxy layers after delamination. The images provide useful information to reconstruct the macroscopic fracture patterns in the *untreated ABS series*. The carbon fibres (Figure 9a), originally black in colour as shown in Figure 7a, are completely covered and hidden by the ABS film, which was light grey in appearance. This suggests that most of the ABS film remained on the carbon/epoxy side of the delaminated surfaces. The ABS film had several small missing parts, easily spotted on the counterpart side of the delaminated surface, i.e., on the glass/epoxy layer (see insets in Figure 9a,b). The missing parts from the carbon/epoxy layer side looked as if they were ripped off by the glass/epoxy layer. Some epoxy protrusions may have grabbed the ABS film generating the black spots on the carbon/epoxy side during the shear deformation of the interface. The appearance of glass/epoxy layer in Figure 9b was more similar to that of the glass/epoxy layer of the baseline series (Figure 7b), although it presented a slightly lighter surface with visible spots of ABS particles attached. The SEM analysis reported in Figure 10a,b confirmed a lower amount of ABS on the glass/epoxy delaminated surface in comparison to the carbon/epoxy layer, which was completely covered by the ABS film. The delaminated surfaces showed a similar rough texture on both separated layers, i.e., carbon/epoxy and glass/epoxy, but with glass fibres clearly exposed. These observations led us to suppose that delamination propagated within the ABS film, but closely to the glass/epoxy layers, as schematically shown in Figure 11a. This hypothesis is also supported by the absence of hackles, which suggests that epoxy was not fractured during the delamination process. The delaminated surfaces appeared rippled, due to the ductile failure of ABS (see insets in Figure 10a,b). 

We further enhanced the stability of delamination by treating the ABS films with oxygen plasma before manufacturing the hybrid laminates. The stress–strain curves related to the *plasma-treated ABS* series are reported in Figure 5c and show a more uniform plateau in the non-linear transition stage than the one observed in the *untreated ABS series*. However, the samples of the plasma-treated *ABS* series delaminated, on average, at lower stress values, (i.e., −18%) than the untreated ABS series. Still, the high scatter in the results makes the comparison of the plasma-treated and untreated series hard. 

Even though the water contact angles confirm the increased hydrophilicity in the ABS surfaces after plasma treatments, the XPS analysis have detected no O-C=O or O-C-O-O functional groups introduced by plasma treatment. In particular, these functional groups possess the ability to perform nucleophilic attacks on the epoxy rings of the epoxy resin monomer, thereby forming covalent bonds between ABS and epoxy. The mechanical test results confirmed that the plasma treatment of the ABS films did not improve adhesion to the composite plies significantly. In fact, the nature of delamination propagation was similar to that in the untreated ABS configuration, i.e., slow and symmetric. The *plasma-treated ABS series* did not present stress drops in the transition stage, generating a stable and uniform plateau although the stability may be related to the lower stress at which the delamination started to propagate. We suspect that the surface treatment in the plasma reactor may have degraded the ABS, reducing the original shear strength or toughness of the material with a consequent lower resistance of ABS against shear deformations. Degradation would explain the lower delamination stress (and *G_IIC_*_,_ too). Then, the stable damage process was not caused by enhanced interface properties, but by the lower energy stored in the samples making sudden drops less probable to occur. 

The delamination patterns of the carbon and glass fibre reinforced layers were analysed after full separation. The analysis with the digital microscope is reported in images (c)–(f) in Figure 9. The appearance of the delaminated surfaces is more uniform than that of the untreated ABS series, i.e., no missing zones due to ABS rips. The ABS film was uniformly attached to the carbon/epoxy layer (see image (c)) leaving the light grey colouring on the surface. The appearance of the glass/epoxy layer (image (d)) was uniform as well, without large pieces of ABS film on the surface. The comparison between the plasma-treated and untreated ABS series in Figure 9 shows a homogeneous distribution of ABS on the glass/epoxy layer of the plasma-treated case. 

Figure 10c,d shows the SEM images. Although they did not show a significant difference between the plasma-treated and untreated samples after delamination, the glass/epoxy layer in Figure 10d showed a slightly higher presence of ABS phase covering the glass fibres of the plasma-treated ABS configuration. The delamination path is believed to be shifted slightly towards the ABS layer (see Figure 11), which may explain why more ABS remained on the glass/epoxy layer after separation. Nevertheless, this observation was hard to quantify and generalise to the whole test series. Figure 11b schematically shows the supposed delamination path, which has left more ABS on the glass/epoxy layer of the plasma-treated ABS series.

#### 3.3.3. Hybrid Composites Containing PS Film Interleaves

**Figure 12:**
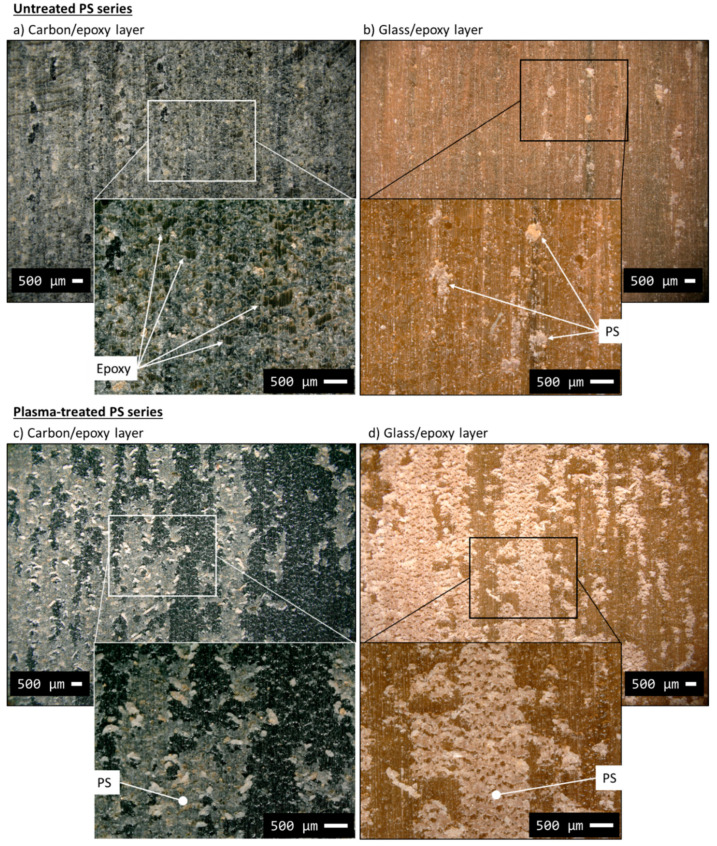
Delaminated surfaces of typical samples from the PS ((**a**,**b**)) and plasma-PS ((**c**,**d**)) series analysed with digital microscope.

The stress–strain curves of the hybrid composites containing PS film interleaves are plotted in Figure 5d. The results summarised in Table 6 show a 23.5% lower delamination stress than that of the baseline, and a *G_IIC_* equal to 0.879 kJ/m^2^ (−41% compared to the baseline). The tensile stress–strain curves showed an initial linear part followed by a transition stage which started to deflect the curve smoothly at around 1% strain. The deflection was caused by a growing delamination around the cut-line in the carbon/epoxy layer. Sudden and catastrophic delamination followed the smooth transition from the initial linear stage, causing multiple stress drops up to complete delamination. The presence of intense stress drops like the ones observed in the baseline series may indicate low resistance of the PS film against delamination or a poor bonding between PS and epoxy. Although delamination propagated at a lower stress than in the baseline, the damage process was unstable anyway. We analysed the carbon/epoxy and glass/epoxy layers after separation using a digital microscope to detect the macroscopic mechanisms promoting early damage propagation in the untreated PS series (Figure 12a,b). The light grey PS film remained mainly attached to the carbon/epoxy layer, but missing pieces of PS are visible as well, which adhered to the glass/epoxy surface (see the inset to Figure 12b). The inset of image (a) shows the presence of epoxy spots surrounded by the failed PS film in detail.

**Figure 13:**
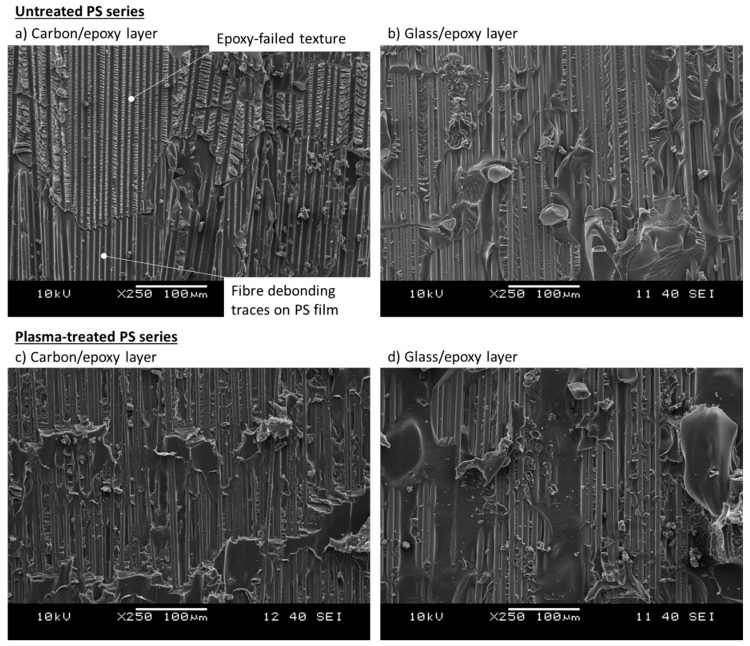
Delaminated surfaces of typical samples from the PS ((**a**,**b**)) and plasma-PS ((**c**,**d**)) series analysed with SEM.

**Figure 14:**
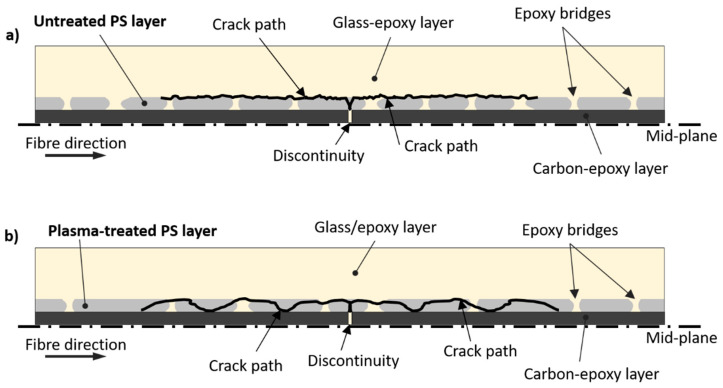
Representation of the crack path in PS-interleaved hybrid composites. The configuration with untreated PS is shown in (**a**) and the configuration with plasma-treated PS in (**b**).

The pieces of epoxy in the PS layer generated distinguishable fracture textures, which were well visible at a lower scale with SEM in Figure 13a. This image includes the different fracture textures we thought were caused by failed epoxy and PS film in the same layer. There were (i) hackles (from failed epoxy) and (ii) smooth surfaces with traces of fibre debonding (from PS film). The hackles were similar to the ones observed in the delaminated samples of the baseline configuration (Figure 7), related to the failed epoxy matrix. The smoother surfaces presenting traces of fibre debonding most probably belonged to the PS film. Taking into account the *T_g_* of PS (i.e., 87 °C), it is reasonable to suppose that the PS films softened significantly at the curing temperature of the composite prepregs (i.e., 125 °C) and let the liquid epoxy penetrate and link the carbon/epoxy and the glass/epoxy layers, creating “epoxy bridges” through the PS interleaf. Since we observed mainly fibre debonding traces on the PS film and a scarce presence of failed PS parts on the delaminated surfaces, we thought that the epoxy bridges played an important role in connecting the composite layers through the PS film and providing shear load transfer capability. In fact, the surfaces of the epoxy bridges after delamination presented hackles (traces of shear failure) consistently, as revealed by SEM analysis in Figure 13a. The early delamination, and low $\sigma_{del}$, registered during the tensile tests of the untreated PS series indicates that the adhesion between PS and epoxy was insufficient. The hypothesised delamination path for this configuration is illustrated in Figure 14a, including also the epoxy bridges in the interleaf layers. The path of delamination is thought to be at the border between the glass/epoxy layer and the PS films as there were no carbon fibres visible in the SEM images on the carbon/epoxy layer, which were completely covered by the PS/epoxy layer. On the contrary, the well visible glass fibres in the glass/epoxy layer after delamination (Figure 13b), supported the hypothesis of a delamination path between glass fibres and the PS/epoxy layer.

Treating PS with oxygen plasma generated better results in comparison with the untreated PS series, in line with our expectations based on the results of the XPS study. The stress–strain curves related to the tensile tests are plotted in Figure 5e and show higher delamination stress (+18% in comparison with the untreated PS configuration) and, consequently, a higher *G_IIC_* (+40% compared to the PS configuration). The initial linear stage terminated with a deflection to form a smooth knee in the stress–strain curves, similar to the one observed in the *untreated PS series*. The initial linear stage was followed by a quasi-stable non-linear transition stage, with low-intensity stress drops compared to those of the *untreated PS series*. This improvement may be attributed to the more reactive PS surface after the oxygen plasma treatment. The treatment increased the polarity of the PS surface, as shown by contact angle tests, and its chemical compatibility with epoxy.

The two series, i.e., *plasma-treated PS and untreated* PS, were compared with microscope analysis in Figure 12 and Figure 13. The macroscopic patterns of delamination in the *plasma-treated PS series* were analysed with the digital microscope (Figure 12c,d). We observed an equal leftover of PS film on both separated surfaces (i.e., carbon/epoxy and glass/epoxy layers) after delamination. The appearance of the surfaces after delamination is displayed by the insets in Figure 12c,d. The structured cracks observed in the PS layer on Figure 12d suggests that it underwent high shear deformation before failure, which may have contributed to better mechanical performance. The fact that the delamination cracks moved into the interleaf layer and pieces of PS were attached to both surfaces confirmed the better adhesion between PS and epoxy, probably promoted by the plasma treatment. The two PS series presented similar features in the tensile stress–strain curves, e.g., stress drops, smooth transition to the non-linear stage, but damage initiation stress in the *plasma-treated PS series* was significantly higher. The O-C=O and O-C-O-O functional groups detected by the XPS analysis of the plasma-treated PS films, may have promoted the formation of covalent bonds between PS and epoxy and resulted in improved PS/epoxy adhesion and finally in a higher delamination stress. The images (c) and (d) of the SEM analysis in Figure 13 show that both carbon and glass fibres were well visible after delamination, indicating a more tortuous delamination path than the one seen in the untreated PS series. Possible crack deflections occurred within the PS film, as schematically shown in Figure 14, which could explain the presence of PS on both separated surfaces. This delamination mechanism was in contrast to the observation made on the *untreated PS series*, where delamination mainly propagated between the glass/epoxy and the interleaf layers, without deflections. Crack deflections are the energy dissipation mechanisms which may explain the higher delamination stress in the plasma-treated PS series, in comparison with the untreated configuration. 

## 4. Conclusions

In this paper, we presented the results of a delamination study in standard thickness carbon/S-glass fibre/epoxy hybrid composites and the effect of interleaving thin ABS and PS thermoplastic films with and without plasma treatment, on the delamination patterns. For this purpose, a special laminate architecture made by continuous glass/epoxy and pre-cut carbon/epoxy layers was adopted to promote mode II delamination. The mode II fracture toughness (*G_IIC_*) of the hybrid composite laminates was evaluated. The thermoplastic films were treated in oxygen plasma to enhance their bonding with the fibre-reinforced epoxy layers. The key findings of this paper are summarised below:

- Interleaving ABS films in hybrid composites increased the delamination stress and the corresponding *G_IIC_* of the layer interfaces. The ABS films reduced the sudden delamination-induced stress drops in the tensile stress–strain curves of the samples.
- The more stable delamination patterns in the untreated ABS series compared to that of the baseline was attributed to the observed damage process zones at the delamination crack front, possibly due to plastic deformation of the interleaved ABS films, which reduced the stress concentration by blunting micro-cracks around the Mode II crack tips.
- The plasma-treated ABS series showed stable delamination without exhibiting stress drops in the stress–strain curves possibly due to reduced delamination stresses associated with an overall decrease of performance.
- Untreated PS films impaired the mechanical properties of the hybrid composites and promoted a sudden spread of delamination causing intense stress drops in the tensile stress–strain curves. It was related to the weak adhesion between PS and epoxy.
- The O-C=O and O-C-O-O functional groups, introduced by oxygen plasma treatment and detected by XPS analysis, promoted covalent bonds between the plasma-treated PS films and epoxy, leading to stronger interface adhesion. The better PS/epoxy bonding introduced energy absorption mechanisms, i.e., crack deflection, generating higher delamination stress and *G_IIC_* in comparison to the untreated PS film-interleaved configuration.

Finally, the approaches presented here may be used to stabilise delamination and increase the interlaminar fracture toughness in hybrid composites. This way, interleaving and surface functionalisation with plasma treatment may lead to hybrid composites failing in a pseudo-ductile way. Further development of this work may be based on using thermoplastics with higher shear strength.

## Figures and Tables

**Table 1:** Mechanical properties of the dry fibres and the epoxy resin based on manufacturer’s data.

Dry Fibres	Tensile Modulus	Strain to Failure	Tensile Strength	Density
	[GPa]	[%]	[MPa]	[kg/m^3^]
AGY S-2 Glass	89	5.7	4890	2470
HexTow IM7 Carbon	276	1.9	5516	1780

**Table 2:** Cured ply properties of unidirectional fibre-reinforced prepregs from the manufacturers’ datasheets.

Prepreg Plies	Nominal Fibre Areal Density	Fibre Volume Fraction	Ply Thickness	Strain to Failure	Elastic Modulus
	[g/m^2^]	[-]	[µm]	[%]	[GPa]
AGY S-2 Glass/913 epoxy	190	0.49	153.8	3.1	45.6
IM7 Carbon/913 epoxy	134	0.58	128.4	1.68	163.2

**Table 3:** Physical, mechanical and thermal properties of the thermoplastic interleaves (from the manufacturer’s datasheets).

Thermoplastic Materials	Density	Tensile Stress at Break	Strain to Failure	Tensile Modulus	Glass Transition Temperature ^(a)^
	[g/cm^3^]	[MPa]	[%]	[GPa]	[°C]
Starex^®^ LX0981 ABS	1.05	32.4	17	2.26	103
Empera^®^ 124N PS	1.04	50	2	3.2	87

^(a)^ Determined by differential scanning calorimetry (DSC).

**Table 4:** Measured water contact angle of the studied thermoplastic films (the coefficient of variation (CV) in % is in brackets below the mean values).

Thermoplastic Films	Contact Angle in [deg] after the Indicated Time of Treatment in Oxygen Plasma		
	0 min	2 min	4 min
**ABS**	93	35	33
	(8.3)	(18.8)	(15.8)
**PS**	85	22	0
	(25.6)	(17.5)	(-)

**Table 5:** Elemental composition of untreated and plasma-treated PS and ABS samples before and after surface ablation with low energy Ar clusters.

Thermoplastic Materials	Elemental Composition [Atomic %]					
	(before Argon Ablation)			(after Argon Ablation)		
	C	O	N	C	O	N
ABS	90.8	5.3	3.9	94.1	0.9	5.1
Plasma-treated ABS	78.2	17.3	4.5	93.8	1.3	5
PS	94.2	5.8	-	99.6	0.4	-
Plasma-treated PS	78.7	21.3	-	99.9	0.2	-

**Table 6:** Results of the tensile tests on the hybrid composite material configurations (the CV in % is in brackets below the mean values).

Material Configurations	Measured Thickness of the Samples	Measured Width of the Samples	Parameters Calculated with the Nominal Thickness (0.75 mm)		Parameters Calculated with the Measured Thickness		Mode II Fracture Toughness (*G_IIC_*) ^(a)^
			Initial Elastic Modulus (*E*_0_)	Delamination Stress (*σ_del_*)	Initial Elastic Modulus (*E*_0_)	Delamination Stress (*σ_del_*)	
	[mm]	[mm]	[GPa]	[MPa]	[GPa]	[MPa]	[kJ/m^2^]
Baseline [G_2_/C/G_2_]	0.77	20.5	64.2	844.5	62.4	820.5	1.503
	(2.90)	(0.32)	(3.89)	(2.63)	(4.10)	(1.31)	(5.27)
Untreated ABS [G_2_/ABS/C/ABS/G_2_]	0.89	20.6	67.5	1014.9	57.0	857.7	2.170
	(3.20)	(0.17)	(3.36)	(1.99)	(0.31)	(5.15)	(3.97)
Plasma-treated ABS [G_2_/P-ABS/C/P-ABS/G_2_]	0.83	20.6	66.2	831.8	59.6	747.3	1.475
	(6.70)	(0.26)	(4.78)	(12.60)	(1.97)	(8.09)	(25.37)
Untreated PS [G_2_/PS/C/PS/G_2_]	0.84	20.3	63.5	646.0	56.4	574.0	0.879
	(1.85)	(0.08)	(3.00)	(0.67)	(1.30)	(1.71)	(1.35)
Plasma-treated PS [G_2_/P-PS/C/P-PS/G_2_]	0.86	20.5	64.1	761.5	55.9	663.9	1.228
	(4.32)	(0.13)	(6.53)	(8.36)	(3.16)	(5.34)	(17.04)

^(a)^ Calculated according to Equation (3), excluding the interleaf layers.

## References

[1] Safri S.N.A.B., Sultan M.T.H. (2019). Damage analysis of glass fiber reinforced composites. Durab. Life Predict. Biocomposites Fibre-Reinf. Compos. Hybrid Compos..

[2] Pagano N.J., Schoeppner G.A., Kelly A., Zweben C.B.T.-C.C.M. (2000). 2.13—Delamination of Polymer Matrix Composites: Problems and Assessment. Comprehensive Composite Materials.

[3] Ferreira De Moura M.F.S. (2008). Interlaminar mode II fracture Characterization. Delamination Behaviour of Composites: A Volume in Woodhead Publishing Series in Composites Science and Engineering.

[4] Mochane M.J., Mokhena T.C., Mokhothu T.H., Mtibe A., Sadiku E.R., Ray S.S., Ibrahim I.D., Daramola O.O. (2019). Recent progress on natural fiber hybrid composites for advanced applications: A review. Express Polym. Lett..

[5] Swolfs Y., Verpoest I., Gorbatikh L. (2019). Recent advances in fibre-hybrid composites: Materials selection, opportunities and applications. Int. Mater. Rev..

[6] Swolfs Y., Gorbatikh L., Verpoest I. (2014). Fibre hybridisation in polymer composites: A review. Compos. Part A Appl. Sci. Manuf..

[7] Czél G., Wisnom M.R. (2013). Demonstration of pseudo-ductility in high performance glass/epoxy composites by hybridisation with thin-ply carbon prepreg. Compos. Part A Appl. Sci. Manuf..

[8] Bismarck A., Bacarreza O., Blaker J., Diao H., Grail G., Pimenta S., Robinson P., Shaffer M. Exploring routes to create high performance pseudo-ductile fibre reinforced composites. Proceedings of the ICCM International Conferences on Composite Materials.

[9] Czél G., Jalalvand M., Wisnom M.R. (2016). Design and characterisation of advanced pseudo-ductile unidirectional thin-ply carbon/epoxy-glass/epoxy hybrid composites. Compos. Struct..

[10] Di Boon Y., Joshi S.C. (2020). A review of methods for improving interlaminar interfaces and fracture toughness of laminated composites. Mater. Today Commun..

[11] Sela N., Ishai O. (1989). Interlaminar fracture toughness and toughening of laminated composite materials: A review. Composites.

[12] Salehi M.M., Hakkak F., Sadati Tilebon S.M., Ataeefard M., Rafizadeh M. (2020). Intelligently optimized electrospun polyacrylonitrile/poly(vinylidene fluoride) nanofiber: Using artificial neural networks. Express Polym. Lett..

[13] Koprivova B., Lisnenko M., Solarska-Sciuk K., Prochazkova R., Novotny V., Mullerova J., Mikes P., Jencova V. (2020). Large-scale electrospinning of poly (Vinylalcohol) nanofibers incorporated with platelet-derived growth factors. Express Polym. Lett..

[14] Lomov S.V., Molnár K. (2016). Compressibility of carbon fabrics with needleless electrospun PAN nanofibrous interleaves. Express Polym. Lett..

[15] Hojo M., Matsuda S., Tanaka M., Ochiai S., Murakami A. (2006). Mode I delamination fatigue properties of interlayer-toughened CF/epoxy laminates. Compos. Sci. Technol..

[16] Zeng Y., Liu H.Y., Mai Y.W., Du X.S. (2012). Improving interlaminar fracture toughness of carbon fibre/epoxy laminates by incorporation of nano-particles. Compos. Part B Eng..

[17] Tsai J.L., Huang B.H., Cheng Y.L. (2011). Enhancing fracture toughness of glass/epoxy composites for wind blades using silica nanoparticles and rubber particles. Procedia Eng..

[18] Ozdemir N.G., Zhang T., Aspin I., Scarpa F., Hadavinia H., Song Y. (2016). Toughening of carbon fibre reinforced polymer composites with rubber nanoparticles for advanced industrial applications. Express Polym. Lett..

[19] Wang C., Sun Q., Lei K., Chen C., Yao L., Peng Z. (2020). Effect of Toughening with Different Liquid Rubber on Dielectric Relaxation Properties of Epoxy Resin. Polymers.

[20] Hayes B.S., Seferis J.C. (2002). Influence of Particle Size Distribution of Preformed Rubber on the Structure and Properties of Composite Systems. J. Compos. Mater..

[21] van der Heijden S., Daelemans L., Meireman T., De Baere I., Rahier H., Van Paepegem W., De Clerck K. (2016). Interlaminar toughening of resin transfer molded laminates by electrospun polycaprolactone structures: Effect of the interleave morphology. Compos. Sci. Technol..

[22] Qin Q., Ye J. (2015). Toughening Mechanisms in Composite Materials.

[23] Molnár K., Košt’áková E., Mészáros L. (2014). The effect of needleless electrospun nanofibrous interleaves on mechanical properties of carbon fabrics/epoxy laminates. Express Polym. Lett..

[24] Gibson A.G. (2000). Continuous Molding of Thermoplastic Composites. Comprehensive Composite Materials.

[25] Kishi H., Nakao N., Kuwashiro S., Matsuda S. (2017). Carbon fiber reinforced thermoplastic composites from acrylic polymer matrices: Interfacial adhesion and physical properties. Express Polym. Lett..

[26] Adeniyi A., Agboola O., Sadiku E.R., Durowoju M.O., Olubambi P.A., Babul Reddy A., Ibrahim I.D., Kupolati W.K. (2016). Thermoplastic-Thermoset Nanostructured Polymer Blends. Des. Appl. Nanostructured Polym. Blends Nanocomposite Syst..

[27] Evans R., Masters J., RE Evans J.E.M., Johnston N.J. (1987). A New Generation of Epoxy Composites for Primary Structural Applications: Materials and Mechanics. Toughened Composites.

[28] Anthony D.B., Bacarreza Nogales O.R., Shaffer M.S.P., Bismarck A., Robinson P., Pimenta S. Pseudo-ductile failure mechanism introduced into finger jointed thermoplastic PES interleaved CFRC. Proceedings of the ECCM 2018—18th European Conference on Composite Materials.

[29] Yun N.G., Won Y.G., Kim S.C. (2004). Toughening of carbon fiber/epoxy composite by inserting polysulfone film to form morphology spectrum. Polymer.

[30] Naffakh M., Dumon M., Gérard J.F. (2006). Study of a reactive epoxy–amine resin enabling in situ dissolution of thermoplastic films during resin transfer moulding for toughening composites. Compos. Sci. Technol..

[31] Shivakumar K., Panduranga R. (2013). Interleaved polymer matrix composites—A review. Proceedings of the 54th AIAA/ASME/ASCE/AHS/ASC Structures, Structural Dynamics, and Materials Conference.

[32] Qian X., Kravchenko O.G., Pedrazzoli D., Manas-Zloczower I. (2018). Effect of polycarbonate film surface morphology and oxygen plasma treatment on mode I and II fracture toughness of interleaved composite laminates. Compos. Part A Appl. Sci. Manuf..

[33] Grail G., Pimenta S., Pinho S.T., Robinson P. (2015). Exploring the potential of interleaving to delay catastrophic failure in unidirectional composites under tensile loading. Compos. Sci. Technol..

[34] Bilge K., Papila M. (2015). Interlayer toughening mechanisms of composite materials. Toughening Mechanisms in Composite Materials.

[35] Wolf R., Sparavigna A.C. (2010). Role of Plasma Surface Treatments on Wetting and Adhesion. Engineering.

[36] Yasuda H., Iriyama Y. (1989). Plasma Polymerization. Compr. Polym. Sci. Suppl..

[37] Hegemann D., Brunner H., Oehr C. (2003). Plasma treatment of polymers for surface and adhesion improvement. Nucl. Instrum. Methods Phys. Res. Sect. B Beam Interact. Mater. Atoms.

[38] Bismarck A. (2015). Springer Wettability: Plasma Treatment Effects. Encyclopedia of Surface and Colloid Science.

[39] Sim K.-B., Baek D., Shin J.-H., Shim G.-S., Jang S.-W., Kim H.-J., Hwang J.-W., Roh J.U. (2020). Enhanced Surface Properties of Carbon Fiber Reinforced Plastic by Epoxy Modified Primer with Plasma for Automotive Applications. Polymers.

[40] Lu C., Qiu S., Lu X., Wang J., Xiao L., Zheng T., Wang X., Zhang D. (2019). Enhancing the Interfacial Strength of Carbon Fiber/Poly(ether ether ketone) Hybrid Composites by Plasma Treatments. Polymers.

[41] Pegoretti A., Karger-Kocsis J. (2018). Interleaving in structural composites: Adapting an old concept to new challenges. Express Polym. Lett..

[42] Zaplotnik R., Vesel A. (2020). Effect of VUV radiation on surface modification of polystyrene exposed to atmospheric pressure plasma jet. Polymers.

[43] Parameswaranpillai J., Hameed N., Pionteck J., Woo E.M. (2017). Handbook of Epoxy Blends|Jyotishkumar Parameswaranpillai.

[44] Jalalvand M., Czél G., Wisnom M.R. (2015). Damage analysis of pseudo-ductile thin-ply UD hybrid composites—A new analytical method. Compos. Part A Appl. Sci. Manuf..

[45] Czél G., Jalalvand M., Wisnom M.R., Czigány T. (2017). Design and characterisation of high performance, pseudo-ductile all-carbon/epoxy unidirectional hybrid composites. Compos. Part B Eng..

[46] Wisnom M.R. (1992). On the Increase in Fracture Energy with Thickness in Delamination of Unidirectional Glass Fibre-Epoxy with Cut Central Plies. J. Reinf. Plast. Compos..

[47] Cui W., Wisnom M.R., Jones M. (1994). An Experimental and Analytical Study of Delamination of Unidirectional Specimens with Cut Central Plies. J. Reinf. Plast. Compos..

[48] Wang Z., Li Z., He Y., Wang Z. (2011). Study of an Environmentally Friendly Surface Etching System of ABS for Improving Adhesion of Electroless Cu film. J. Electrochem. Soc..

[49] Paynter R.W. (1998). XPS studies of the modification of polystyrene and polyethyleneterephthalate surfaces by oxygen and nitrogen plasmas. Surf. Interface Anal..

[50] Wisnom M.R., Czél G., Swolfs Y., Jalalvand M., Gorbatikh L., Verpoest I. (2016). Hybrid effects in thin ply carbon/glass unidirectional laminates: Accurate experimental determination and prediction. Compos. Part A Appl. Sci. Manuf..

[51] Rev T., Jalalvand M., Fuller J., Wisnom M.R., Czél G. (2019). A simple and robust approach for visual overload indication—UD thin-ply hybrid composite sensors. Compos. Part A Appl. Sci. Manuf..

[52] Greenhalgh E.S., Hiley M.J. Fractography of Polymer Composites: Current Status and Future Issues. Proceedings of the 13th European Conference on Composite Materials.

[53] Greenhalgh E.S. (2009). Failure Analysis and Fractography of Polymer Composites.

